# Calcium Circadian Rhythmicity in the Suprachiasmatic Nucleus: Cell Autonomy and Network Modulation

**DOI:** 10.1523/ENEURO.0160-17.2017

**Published:** 2017-08-18

**Authors:** Takako Noguchi, Tanya L. Leise, Nathaniel J. Kingsbury, Tanja Diemer, Lexie L. Wang, Michael A. Henson, David K. Welsh

**Affiliations:** 1 Department of Psychiatry and Center for Circadian Biology, University of California, San Diego, La Jolla, CA 92093-0603; 2 Department of Mathematics and Statistics, Amherst College, Amherst, MA 01002; 3 Department of Chemical Engineering and Institute of Applied Life Sciences, University of Massachusetts Amherst, Amherst, MA 01003-9364; 4 Veterans Affairs San Diego Healthcare System, San Diego, CA 92161

**Keywords:** Calcium imaging, circadian rhythm, luciferase imaging, PER2, suprachiasmatic nucleus

## Abstract

Circadian rhythms of mammalian physiology and behavior are coordinated by the suprachiasmatic nucleus (SCN) in the hypothalamus. Within SCN neurons, various aspects of cell physiology exhibit circadian oscillations, including circadian clock gene expression, levels of intracellular Ca^2+^ ([Ca^2+^]_i_), and neuronal firing rate. [Ca^2+^]_i_ oscillates in SCN neurons even in the absence of neuronal firing. To determine the causal relationship between circadian clock gene expression and [Ca^2+^]_i_ rhythms in the SCN, as well as the SCN neuronal network dependence of [Ca^2+^]_i_ rhythms, we introduced GCaMP3, a genetically encoded fluorescent Ca^2+^ indicator, into SCN neurons from PER2::LUC knock-in reporter mice. Then, PER2 and [Ca^2+^]_i_ were imaged in SCN dispersed and organotypic slice cultures. In dispersed cells, PER2 and [Ca^2+^]_i_ both exhibited cell autonomous circadian rhythms, but [Ca^2+^]_i_ rhythms were typically weaker than PER2 rhythms. This result matches the predictions of a detailed mathematical model in which clock gene rhythms drive [Ca^2+^]_i_ rhythms. As predicted by the model, PER2 and [Ca^2+^]_i_ rhythms were both stronger in SCN slices than in dispersed cells and were weakened by blocking neuronal firing in slices but not in dispersed cells. The phase relationship between [Ca^2+^]_i_ and PER2 rhythms was more variable in cells within slices than in dispersed cells. Both PER2 and [Ca^2+^]_i_ rhythms were abolished in SCN cells deficient in the essential clock gene *Bmal1*. These results suggest that the circadian rhythm of [Ca^2+^]_i_ in SCN neurons is cell autonomous and dependent on clock gene rhythms, but reinforced and modulated by a synchronized SCN neuronal network.

## Significance Statement

Intracellular calcium ([Ca^2+^]_i_) oscillates with a circadian (∼24-h) rhythm within the SCN, the brain’s master circadian pacemaker. However, what drives these [Ca^2+^]_i_ rhythms is not well understood. To test how rhythmic circadian clock gene expression and the SCN neuronal network influence [Ca^2+^]_i_ rhythms, we monitored [Ca^2+^]_i_ and PER2 (a rhythmic clock gene) in individual SCN neurons in dispersed and slice cultures by fluorescence and bioluminescence imaging, respectively. We also tested the effects of blocking neuronal firing or knocking out *Bmal1*, an essential clock gene. We found that the [Ca^2+^]_i_ rhythm requires intact clock gene rhythms, and that it does not require the SCN neuronal network (so is cell autonomous) but is reinforced and modulated by the neuronal network.

## Introduction

In mammals, circadian rhythmicity of behavior and physiology is controlled by a master circadian pacemaker in the hypothalamus: the suprachiasmatic nucleus (SCN). Circadian rhythm generation within SCN neurons and other cells depends on a core transcription-translation negative feedback loop (TTFL). This core loop involves activation of *Period* (*Per1*, *Per2*, *Per3*) and *Cryptochrome* (*Cry1* and *Cry2*) gene transcription by a BMAL1/CLOCK heterodimer, and delayed inhibition of this process by complexes containing PER and CRY proteins (Mohawk and Takahashi, 2011). Null mutation of *Bmal1* alone is sufficient to abolish circadian rhythms of behavior (Bunger et al., 2000) or single SCN neurons (Ko et al., 2010).

In SCN neurons, various cellular processes exhibit circadian rhythms, including clock gene expression, Ca^2+^, neuronal firing rate, and neuropeptide release (Welsh et al., 2010). SCN neurons communicate through synapses (Yamaguchi et al., 2003), diffusible messengers (Silver et al., 1996; Maywood et al., 2011), and possibly gap junctions (Colwell, 2000b) to create coherent rhythms. Although individual SCN neurons can function as independent circadian oscillators (Welsh et al., 1995), the SCN network contributes to the strength of cellular rhythmicity (Webb et al., 2009).

Ca^2+^ plays important roles in both generation of circadian rhythms in SCN neurons and their synchronization by retinal input (Colwell, 2011). Previous studies found adequate extracellular and intracellular Ca^2+^ levels to be essential for generation and regulation of cellular circadian rhythms (Lundkvist et al., 2005; Harrisingh et al., 2007). In retinorecipient SCN neurons, [Ca^2+^]_i_ is also an important mediator in the signaling pathway from postsynaptic glutamate receptors to clock gene expression (Colwell, 2011).

Circadian oscillation of [Ca^2+^]_i_ has been demonstrated in plants (Johnson et al., 1995), *Drosophila* (Liang et al., 2016), and rodent SCN (Colwell, 2000a; Ikeda et al., 2003; Enoki et al., 2012; Brancaccio et al., 2013). One early study using the Ca^2+^-sensitive dye fura2 (Colwell, 2000a) found a circadian [Ca^2+^]_i_ rhythm that is dependent on both neuronal firing and voltage-gated Ca^2+^ channel activity. In contrast, studies using genetically encoded Ca^2+^ reporters have revealed [Ca^2+^]_i_ rhythms that persist in the absence of neuronal firing (Enoki et al., 2012; Brancaccio et al., 2013) but are suppressed by blockers of ryanodine-sensitive intracellular [Ca^2+^] stores (Ikeda et al., 2003). Although the relationship between clock gene and [Ca^2+^]_i_ rhythms has not been studied at a single-cell level in dispersed culture, [Ca^2+^]_i_ peak times precede PER2 peak times in SCN slices (Brancaccio et al., 2013; Enoki et al., 2017a) and show a spatiotemporal wave pattern similar to that previously reported for *Per1* and PER2 (Yamaguchi et al., 2003; Foley et al., 2011; Enoki et al., 2012). In *Cry1*/*Cry2* double knockout mice, SCN [Ca^2+^]_i_ and neuronal firing rhythms are abolished, especially in dorsal SCN (Enoki et al., 2017a). Phase relationships between [Ca^2+^]_i_ and neuronal firing rhythms are different in dorsal and ventral mouse SCN (Enoki et al., 2017b). In *Drosophila*, the [Ca^2+^]_i_ rhythm is attenuated in *per^01^* null mutant flies, which lack molecular and behavioral rhythmicity, and its phase is modulated by neuropeptide signaling (Liang et al., 2016). These studies raise intriguing questions about how the SCN network regulates [Ca^2+^]_i_–PER2 phase relationships spatiotemporally, and how [Ca^2+^]_i_ rhythms are related to neuronal firing, neuronal networks, and other important clock genes.

In this study, we evaluated three possible mechanisms that might contribute to SCN [Ca^2+^]_i_ rhythms: (1) the TTFL molecular clock, (2) spontaneous action potentials within single SCN neurons, and (3) the synchronized neuronal network coupled by neurotransmitters. To monitor [Ca^2+^]_i_ and clock gene expression rhythms simultaneously in individual SCN neurons, the genetically encoded Ca^2+^ reporter GCaMP3 was introduced by adeno-associated virus (AAV) to SCN slices or dispersed cells cultured from PER2::LUC knock-in reporter mice, which express a fusion of PER2 and firefly luciferase in all cells (Welsh et al., 2004). Furthermore, we used computational models and image analysis to reveal neuronal network contributions to the generation and spatiotemporal patterning of clock gene and [Ca^2+^]_i_ rhythms.

## Materials and Methods

### Experimental design and statistical analysis

#### Animals

Generation of mPer2^Luciferase^ (PER2::LUC) knock-in mice was described previously (Yoo et al., 2004). For this study, we used an alternative PER2::LUC mouse line incorporating an SV40 polyadenylation site to enhance expression levels (Welsh et al., 2004). The mice were developed at Northwestern University using the same methodology as the original strain of knock-in mice. PER2::LUC mice were backcrossed for >10 generations with wild-type (WT) mice (C57BL/6J) purchased from the Jackson Laboratory. *Bmal1* ^–/–^ mice were donated by Dr. Pamela Mellon (University of California, San Diego). This line was created by crossing *Bmal1*-floxed mice (The Jackson Laboratory, stock number 7668; Storch et al., 2007) and *zona pellucida* 3 (Zp3)-*cre* mice (Lewandoski et al., 1997). PER2::LUC;*Bmal1* ^–/–^ mice were created by crossing PER2::LUC mice and *Bmal1* ^–/–^ mice. PER2::LUC mice were bred as homozygotes, and PER2::LUC;*Bmal1* ^–/–^ mice were bred as heterozygotes at the *Bmal1* allele *(Bmal1* ^+/–^). Genotyping was performed by multiplex PCR as described in a previous report (Storch et al., 2007).

Mice were maintained in LD 12:12-h cycles (12 h light, 12 h dark) throughout gestation and from birth until used for experiments. Mouse studies were conducted in accordance with regulations of the Institutional Animal Care and Use Committee at University of California, San Diego.

#### SCN slice and dispersed culture

Both male and female mice were used in this study. For SCN slice cultures, 4- to 7-d-old PER2::LUC knock-in mice or PER2::LUC;*Bmal1* ^–/–^ mice were anesthetized by ketamine (Ketaset, Fort Dodge Animal Health) and decapitated. The hypothalamus block was dissected from the whole brain, and coronal slices were cut by a tissue chopper (Stoelting) to a thickness of 250 µm. Each SCN slice was cultured on a Millicell-CM membrane insert (EMD Millipore PICMORG50; Merck KGaA) in explant medium with low sodium bicarbonate (EML) [DMEM (Thermo Fisher Scientific, 12100046), supplemented with 350 mg/L sodium bicarbonate, 10 mM HEPES, 25 U/mL penicillin, 25 µg/mL streptomycin, 2% B-27 (Thermo Fisher Scientific, 17504-044), and 200 µM d-luciferin potassium salt (BioSynth L-8220; no phenol red, serum-free; pH 7.4].

Dispersed SCN neurons were obtained from neonatal (0- to 4-d-old) PER2::LUC knock-in mice. 1 × 10^5^ cells were plated in a well of a glass bottom dish (MatTek P35G-1.5-10-C) and cultured as described previously (Welsh et al., 1995; Liu et al., 2007). Tetrodotoxin (TTX) and ryanodine were purchased from Sigma-Aldrich (T5651).

#### Viral vector transduction

AAV-2/1-hSynapsin-GCaMP3 was purchased from the vector core laboratory of the University of Pennsylvania. For dispersed cell culture, 3 µl virus solution (5 × 10^11^ genomic copies/0.1 mL) was mixed with dispersed cells (∼200 µl) during the first 2 h of plating. For slice culture, 0.5 µl virus solution was dropped onto an SCN slice immediately after the start of culture. To allow GCaMP3 expression levels to saturate, SCN dispersed cells and slices were cultured for at least 2 wks before imaging. Strong GCaMP3 expression was observed in most neurons.

#### Fluorescence and bioluminescence imaging

Simultaneous fluorescence (FL) and bioluminescence (BL) imaging was performed by adding FL imaging to previously described methods for BL imaging (Welsh et al., 2005; Welsh and Noguchi, 2012). The culture was placed on the stage of a microscope (Olympus IX-71). Light from the sample was collected with a 10× objective lens (10× UPlanSApo; Olympus) and transmitted to a cooled CCD camera (iKon-M; Andor) mounted on the bottom port of the microscope. The image was focused using GCaMP3 fluorescence, lights were turned off, and stray light was eliminated by covering the dish with a small black lucite box and draping the microscope with black plastic sheeting (Thorlabs BK5). GCaMP3 was excited by a 500-nm LED (pE-2; coolLED), and emitted light was filtered by a yellow fluorescent protein (YFP) filter cube (YFP-2427A; Semrock). LED light intensity was reduced to either 10% or 50% of maximal level, depending on GCaMP3 expression levels. FL images were collected at intervals of 30 min or 1 h with 1-s exposure duration, no binning, pre-amplifier gain ×4, and readout speed 5 MHz. To avoid autofluorescence of luciferin, excitation and filter settings were optimized for YFP rather than for green fluorescent protein (GFP), which has absorbance and emission spectra more similar to GCaMP3 (Tian et al., 2009). For BL image collection, the LED light was turned off, and the filter cube turret was moved to an open (no filter cube) position. BL images were collected at intervals of 30 min or 1 h, alternating with FL images, with 29-min exposure duration, binning 1 × 1 or 4 × 4, pre-amplifier gain ×4, and readout speed 50 kHz. To align with FL images, BL images were shifted 1 pixel in the *x* direction and –9 pixels in the *y* direction. MetaMorph (Molecular Devices) was used for control of camera and LED shutter and for image analysis. Time series images were typically collected for 5–7 d.

#### Image processing for manual cell tracking

In MetaMorph, stack files of time series of either FL or BL images were created. To remove cosmic ray artifacts, pairs of consecutive images were compared pixel-wise, and the minimum values of all pixels were used to construct a new image from every pair of consecutive images (running minimum algorithm; Welsh et al., 2004). The same algorithm was run on a FL stack file to make it comparable to a BL stack file. Then, a combined stack file was constructed by interleaving consecutive FL and BL image planes. In dispersed cell cultures, neurons were morphologically identified by their round cell bodies and long processes in FL images, and matching of neurons in alternating FL and BL images ensured identification of single cells. Bright PER2::LUC signals also confirmed that neurons were derived from the SCN. Both FL and BL intensity were measured within a region of interest (ROI) defined manually for each cell. For dispersed cultures, smaller (3 × 3-pixel) ROIs were used for FL images, selecting the brightest part of a cell, and larger (∼10 × 10-pixel) ROIs about the same size as a cell body were used for BL images. In slice cultures, identification of single cells was difficult, especially in BL images with lower resolution, because of high cell density and multiple layers of cells in a slice. For slice cultures, small (3 × 3-pixel) ROIs were used for both FL and BL, selecting the brightest part of a cell in FL images, and measuring the intensity of both FL and BL in the same position in a combined stack file. The position of the ROI was adjusted to accommodate movements of cells during the experiment, but its size was kept constant across the time series and across cells. Data were logged to Microsoft Excel files for plotting and further analysis.

**Figure 1.**
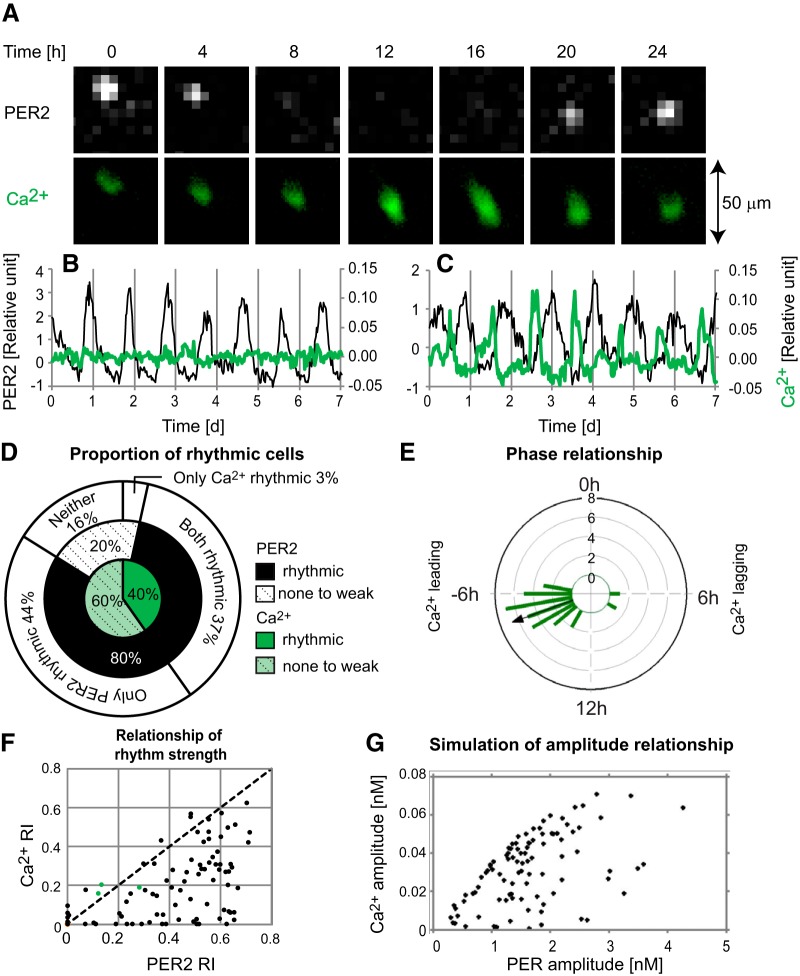
PER2 and [Ca^2+^]_i_ dynamics of single dispersed SCN neurons. ***A***, Time-lapse images of PER2 and [Ca^2+^]_i_ imaged simultaneously in a single neuron. Time 0 is 4.3 d after start of imaging. PER2 and [Ca^2+^]_i_ were reported by bioluminescence intensity of PER2::LUC and fluorescence intensity of GCaMP3, respectively. ***B***, ***C***, Representative patterns of PER2 and [Ca^2+^]_i_ in single neurons. Shown are relative PER2 expression (black line, left axis) and [Ca^2+^]_i_ (green line, right axis). Time 0 is start of imaging. Shown are a cell with a clear PER2 rhythm, but no [Ca^2+^]_i_ rhythm (***B***) and a cell with rhythmic PER2 and [Ca^2+^]_i_ (***C***). Values are calculated by the procedures described in Materials and Methods as well as in Fig. 1-1. Further examples of single cell traces are shown in Fig. 1-2. ***D***, Percentages of cells categorized as having rhythmic PER2 or [Ca^2+^]_i_. Black and green portions show proportions of cells with clearly rhythmic PER2 and [Ca^2+^]_i_, respectively. Stippled white and light green portions show proportions of cells with nonrhythmic (or very weakly rhythmic) PER2 and [Ca^2+^]_i_, respectively. Numbers were rounded to the nearest 1%. ***E***, A Rayleigh histogram showing differences between PER2 and [Ca^2+^]_i_ rhythm peak time for individual neurons. Negative or positive values indicate that [Ca^2+^]_i_ peak is leading or lagging PER2 peak, respectively. Length of bars indicates number of cells within each 1-h bin (*n* = 32 cells in 3 cultures). Arrow indicates a mean vector. ***F***, Relationship between PER2 and [Ca^2+^]_i_ RI in dispersed cells, with [Ca^2+^]_i_ RI plotted against PER2 RI of the same cell (black dots). Green dots are exceptional cells categorized as rhythmic for [Ca^2+^]_i_ but not PER2. The black dotted line is a guide line where PER2 and [Ca^2+^]_i_ RI are equal. ***G***, Simulation of the relationship between PER and [Ca^2+^]_i_ amplitude in a mathematical model. [Ca^2+^]_i_ amplitude is limited by PER amplitude.

**Figure 2.**
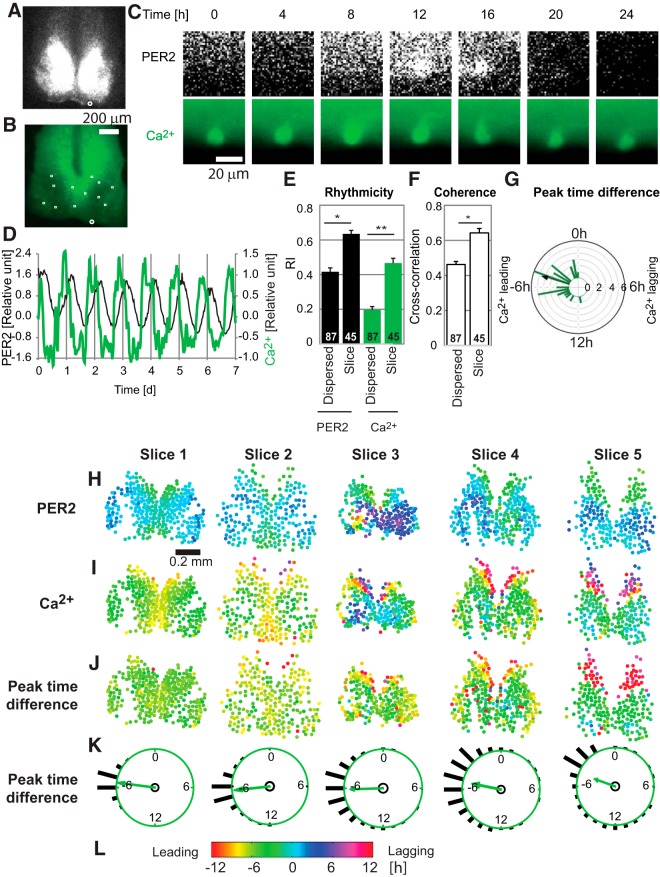
PER2 and [Ca^2+^]_i_ dynamics of single neurons in SCN slices. Representative images of PER2 (***A***) and [Ca^2+^]_i_ (***B***) in an SCN slice. Positions of cells selected for data analysis were marked by white squares and a circle. ***C***, Time-lapse images (at 4-h intervals) for the 50 × 50-µm area marked by the white circle in ***A*** and ***B***. Time 0 is 3.7 d after start of imaging. ***D***, Representative patterns of PER2 (black lines, left axis) and [Ca^2+^]_i_ (green lines, right axis) for a single cell within an SCN slice, showing clear PER2 and [Ca^2+^]_i_ rhythms. Time 0 is start of imaging. ***E***, ***F***, Comparisons of single SCN neurons in dispersed versus slice cultures, showing PER2 and [Ca^2+^]_i_ rhythmicity (RI) (***E***) and coherence between PER2 and [Ca^2+^]_i_ rhythms (***F***). Values are averages ± SEM, with numbers of cells shown on bars. *, *p* < 0.05; **, *p* < 0.01, mixed effect model. ***G***, Rayleigh histogram showing the distribution of differences between PER2 and [Ca^2+^]_i_ peak times for individual cells in SCN slices. Negative or positive values indicate that [Ca^2+^]_i_ peak is leading or lagging PER2 peak, respectively. Length of bars indicates number of cells within each 1-h bin (*n* = 32 cells in three slices). Arrow indicates a mean vector. ***H–L***, Spatiotemporal relationships between PER2 and [Ca^2+^]_i_ peaks of five SCN slices. ***H***, ***I***, For each of the five slices, cell-like regions with significant rhythmicity in both PER2 and [Ca^2+^]_i_ are plotted as circles, with peak phases color-coded as in ***L***. PER2 (***H***) or [Ca^2+^]_i_ (***I***) peak times are shown relative to the PER2 peak time of the whole slice, with negative values indicating phase leading of cell-like regions. ***J***, Peak time differences between PER2 and [Ca^2+^]_i_ are also shown, color-coded as in ***L***. Negative values indicating phase leading of [Ca^2+^]_i_ peaks relative to PER2 peaks in the same cell-like regions. ***K***, Rayleigh histograms show corresponding distributions of these peak time differences. Numbers of cell-like regions: 267, 238, 149, 331, and 179 in slices 1–5, respectively. Bar length indicates the proportion of cells in each bin. Arrows indicate mean vectors, all of which extend outside the small inner circles marking criterion levels for statistical significance (Rayleigh test, *p* < 0.01), indicating that the distributions are all significantly different from uniform.

**Figure 3.**
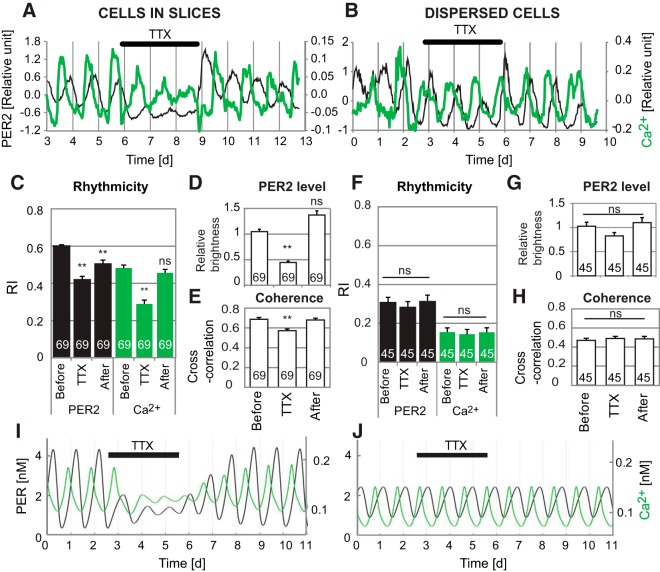
Effects of TTX on SCN neurons in slice and dispersed cultures. Representative patterns of PER2 and [Ca^2+^]_i_ for a cell in an SCN slice (***A***) and a dispersed cell (***B***). Each plot shows relative levels of PER2 expression (black lines, left axis) and [Ca^2+^]_i_ (green lines, right axis) for a single SCN neuron. Time 0 is start of imaging. Black bar indicates duration of TTX application. In SCN slices, PER2 expression and rhythmicity decreased during TTX in all cells, and [Ca^2+^]_i_ rhythmicity also decreased significantly on average, whereas in dispersed cells TTX had no significant effects. Shown here, ***A*** is a cell in a slice for which the PER2 rhythm damped substantially and the [Ca^2+^]_i_ rhythm damped more modestly during TTX, and ***B*** is a dispersed cell in which TTX had no discernible effect on either rhythm. ***C–H***, Bar graphs of RI (***C***, ***F***), PER2 expression (***D***, ***G***), and coherence between PER2 and [Ca^2+^]_i_ rhythms (***E***, ***H***) before, during, and after TTX application, for cells in slices and dispersed cells, respectively. All values shown are averages ± SEM, with numbers of cells shown on bars. **, *p* < 0.01; ns, not significant (mixed effect model compared to before TTX application). Effects of ryanodine are shown in Fig. 3-1. ***I***, ***J***, Simulations of PER expression (black lines, left axis) and [Ca^2+^]_i_ (green lines, right axis) for a single cell in a multicellular model (***I***) and a single-cell model (***J***). Black bars indicate duration of simulated TTX application. In the multicellular model, PER and [Ca^2+^]_i_ levels and rhythmicity decreased during TTX, whereas in the single-cell model, TTX had no effect.

**Figure 4.**
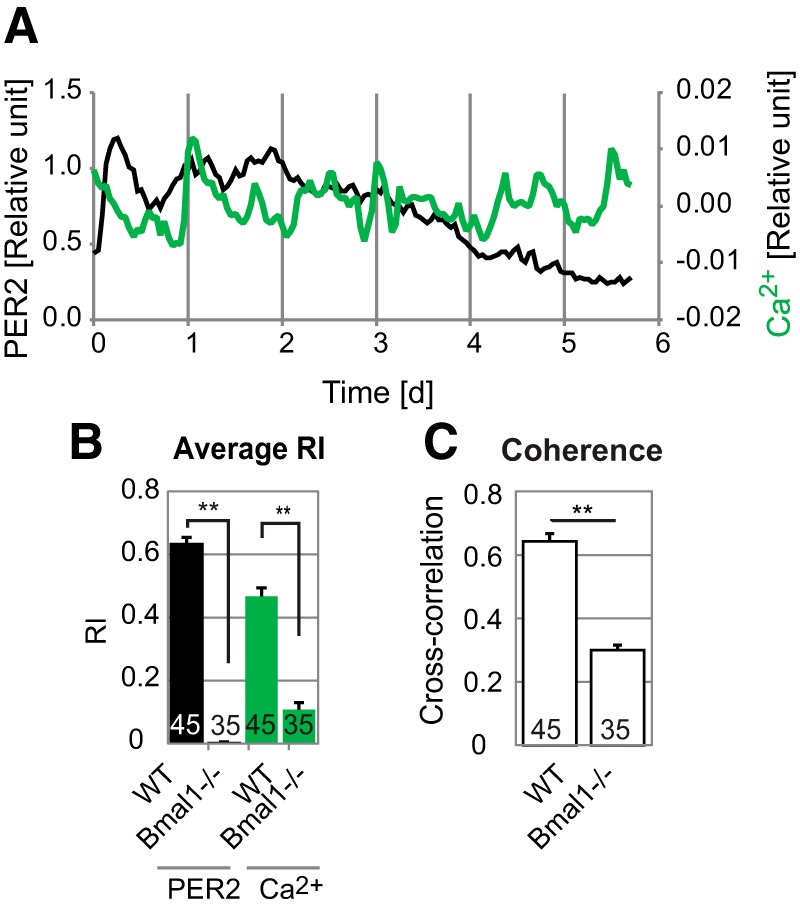
***A***, PER2 and [Ca^2+^]_i_ patterns of a representative cell in a *Bmal1^–/–^* SCN slice culture. Relative levels of PER2 (black lines, left axis) and [Ca^2+^]_i_ (green lines, right axis) are shown. Time 0 is start of imaging. ***B***, Comparison of average RI values for PER2 rhythms (black bars) and [Ca^2+^]_i_ rhythms (green bars) for cells in WT and *Bmal1^–/–^* SCN slices. ***C***, Coherence between PER2 and [Ca^2+^]_i_ rhythms. **, *p* < 0.01, *t* test. All values shown are averages ± SEM, with numbers of cells shown on bars.

BL intensity values were corrected for background level in each experiment by subtracting a single minimum intensity of a background region devoid of cells. Background-subtracted values were normalized to an average BL intensity (PER2 level) of neurons in the same dish. After subtracting 1 from each normalized value, the resulting value was plotted in relative units (i.e., 0 = average PER2 level of neurons in the dish; Figs. 1*B*, *C*, 2*D*, 3*A*, *B*, and 4*A*). See figures in the Results section.

10.1523/ENEURO.0160-17.2017.f1-l Figure 1-1 Normalization of FL intensity of cells using background. (A) Example of a whole field of view of [Ca^2+^]_i_ reported by GCaMP3. Note the considerable variation across the image in intensity of background areas devoid of cells (arrow heads 1–6). (B) Examples of fluctuation of background intensity over time, showing intensities of background (BG) areas indicated by arrow heads in A. CCD camera and software had to be reset at ∼5.7 d after start of imaging. (C-D) Linear correlation between intensity of BGn and coefficient An (C) or constant Bn (D) (for details, see Materials and Methods). An and Bn values for background regions (blue diamonds) could be used to extrapolate corresponding values for cell-containing regions of higher intensities (Acell, Bcell, magenta diamond), and from these to calculate an expected background intensity value for each cell. (E-F) Patterns of estimated background (blue) and raw FL intensity (black) for two representative cells, one non-rhythmic (E, cell1) and the other rhythmic (F, cell2). (G) Ratios of raw FL intensity to expected BG for cell1 (black) and cell2 (green). (H) Ratios shown in G after detrending by subtracting a 24 h running average. Download Figure 1-1, EPS file.

10.1523/ENEURO.0160-17.2017.f1-2 Figure 1-2 Additional plots of PER2 (black lines, left axis) and [Ca^2+^]_i_ (green lines, right axis) for SCN cells exhibiting various patterns of [Ca^2+^]_i_. Shown at left are cells in dispersed cultures (A-E), including a cell with a sinusoidal [Ca^2+^]_i_ rhythm (A), a cell with a [Ca^2+^]_i_ rhythm showing a secondary peak (B), an initially non-rhythmic cell with spontaneous recovery of both PER2 and [Ca^2+^]_i_ rhythms (C), and cells in which the [Ca^2+^]_i_ rhythm became weaker (D) or stronger (E) during TTX. Shown at right are cells in SCN slice cultures (F-J), including a cell with a sinusoidal [Ca^2+^]_i_ rhythm (F), a cell with a [Ca^2+^]_i_ rhythm showing a secondary peak (G), a cell with an unusually phased [Ca^2+^]_i_ rhythm peaking after PER2 (H), a cell in which TTX had no discernible effect on the [Ca^2+^]_i_ rhythm (I), and a cell in which the [Ca^2+^]_i_ rhythm was weaker during TTX (J). Download Figure 1-2, EPS file.

10.1523/ENEURO.0160-17.2017.f3-1 Figure 3-1 Effects of ryanodine on PER2 and [Ca^2+^]_i_ rhythm in dispersed SCN cells. (A) PER2 and [Ca^2+^]_i_ patterns of a representative cell in a dispersed cell culture. Relative levels of PER2 (black lines, left axis) and [Ca^2+^]_i_ (green lines, right axis) are shown. Time 0 is start of imaging. (B) Comparison of average RI values for PER2 rhythms (black bars) and [Ca^2+^]_i_ rhythms (green bars) for cells before and during 100 μM ryanodine application. n.s. *p* > 0.05, mixed effect model. Download Figure 3-1, EPS file.

FL intensity of background regions devoid of cells varied with position and time, reflecting variations in the LED light source, CCD camera operation, and a slow upward trend in GCaMP3 expression over time. Therefore, FL intensity of a cell was normalized using a set of multiple background regions devoid of cells, and then detrended by subtracting a 24-h running average. In each dissociated cell experiment, six different ROIs in areas devoid of cells were selected as backgrounds. For slice cultures, nonrhythmic tissue areas surrounding SCN were included as background ROIs. Time series were plotted of the intensities of these six backgrounds (BG_1_ to BG_6_, named from low to high intensity). At each time point, we assumed that intensities of these background regions were linearly related:

$$BG_{n}=A_{n}\times BG_{6}+B_{n},$$

where *BG_n_* are background intensity values, *A_n_* are coefficients, and *B_n_* are constants. *A_n_* and *B_n_* (*n* = 1, 2, 3, 4, 5) could then be obtained using *BG_n_* values at two time points (minimum and maximum values in the time series). Both *A_n_* and *B_n_* (*n* = 1, 2, 3, 4, 5) were linearly related to the average intensity of *BG_n_*. Using these linear relationships, *A_cell_* and *B_cell_* could then be obtained for any cell-containing region with a particular average intensity, and the expected background intensity for each cell (*BG_cell_*) estimated by the following equation:

$$BG_{cell}=A_{cell}\times BG_{6}+B_{cell}.$$

Thus, FL intensity of each cell at each time point was normalized to an expected background intensity by dividing by *BG_cell_*, and long-term trend was removed by subtracting a 24-h running average. For data in the first or last 12 h, the first or last 24-h average was subtracted, respectively. Processed FL intensity ([Ca^2+^]_i_) values are shown on the right axes of data plots (Figs. 1*B*, *C*, 2*D*, 3*A*, *B*, and 4*A*).

#### Rhythm data analysis for manually tracked data

Single-cell circadian rhythmicity was assessed in two ways. Autocorrelation was applied as a simple and robust metric of rhythmicity, using xcorr in Matlab (MathWorks). This method has the advantage of being independent of waveform, so it is equally applicable to PER2 rhythms (which resemble a sine wave) and [Ca^2+^]_i_ rhythms (which do not). BL and FL data were processed as described above, and then the autocorrelation was calculated for 3- or 4-d segments of each time series after linear detrending. The rhythmicity index (RI) was defined as the value of autocorrelation at 24-h lag, which is applicable even for neurons without significant rhythmicity, such as *Bmal1* ^–/–^ neurons. RI varies from 0 to 1, where 1 indicates a perfectly periodic time series. To assess for the presence of statistically significant rhythmicity, we used a test developed previously (Leise et al., 2012) based on regression of the log-log plot of the power spectral density and motivated by the analysis of arrhythmic *Bmal1^–/–^* SCN cells reported by Ko et al. (2010). Here, we call this test the PSD-regression test. Cells with *p* < 0.05 (PSD-regression test) were categorized as rhythmic. Circular statistics were run in Matlab using the CircStat package (Berens, 2009).

In Figs. 1, 2, and 4, 4D segments of time series were used to assess rhythmicity with both RI and the PSD-regression test. In Fig. 3, 3D segments before, during, and after TTX application were used. In control conditions, RI was affected by duration of data (*p* < 0.01, *t* test; Figs. 2*E* and 3*C*, *E*), so for TTX experiments, we ensured that all segments were of similar duration. For cross-correlations in TTX experiments (Fig. 3*E*, *H*), average PER2 intensity over the 2.0 d before TTX application (before), from 0.5 to 2.5 d after TTX application (TTX), and from 0.5 to 2.5 d after washout (after) were used.

For cells categorized as rhythmic, coherence between PER2 and [Ca^2+^]_i_ rhythms was calculated as the peak of cross-correlation for linear-detrended time series using xcorr in Matlab. The time lag associated with this peak was used to estimate the phase difference between the PER2 and [Ca^2+^]_i_ rhythms of each cell. To test for statistical differences of RI and coherence between SCN slice cultures and dispersed cell cultures, Fisher *z*-transformation followed by hierarchical (mixed effect) model (Bates et al., 2015) was applied using R (Bates et al., 2015; Brockhoff et al., 2016).

#### Spatiotemporal imaging data analysis

After removing cosmic rays by a running minimum algorithm in MetaMorph, FL and BL stack files were analyzed using ImageJ and Matlab scripts using methods described previously (Evans et al., 2011a). Briefly, ROIs of cell-like regions were identified as small bright disks in the 2D wavelet filtered sum of days 0.5–2.5 of the recording. Cell-like regions were judged to be significantly rhythmic if they passed the Siegel test at significance level 0.01. Peak times of PER2 and [Ca^2+^]_i_ for each cell-like region were calculated after detrending using a discrete wavelet transform.

### Mathematical models

The effect of TTX on the SCN was studied using previously developed mathematical models of the SCN (Vasalou and Henson, 2010, 2011; Kingsbury et al., 2016). All simulations were performed in Matlab. The model had four basic components: core molecular clock, electrophysiology, network topology, and neurotransmitter signaling. The previous model contained 21 ordinary differential equations (ODEs): the 16 ODEs of the Leloup–Goldbeter core molecular clock model (Leloup and Goldbeter, 2003), two ODEs governing both cytoplasmic ([Ca^2+^]_i_) and ryanodine storage calcium concentrations (Vasalou and Henson, 2010), one ODE for the phosphorylated cAMP response element-binding protein (CREB) concentration (To et al., 2007), and two ODEs for the neurotransmitters vasoactive intestinal peptide (VIP) and γ-aminobutyric acid (GABA), which controlled all cell-to-cell signaling. The relationship between [Ca^2+^]_i_ and the kinetics of CREB phosphorylation, as well as activated CREB’s feedback on the molecular clock, have been modeled previously (To et al., 2007) based on principles from experimental observation (Obrietan et al., 1999; Tischkau et al., 2003).

To generate heterogeneity within the network population, we randomly distributed the parameters determining the baseline rates of *Per* mRNA transcription (*v_sP0_*; 0.94 ± 0.06), *Bmal1* mRNA transcription (*v_sB_*; 1.00 ± 0.01), and *Bmal1* mRNA degradation (*v_mB_*; 0.80 ± 0.01), as described previously (Vasalou et al., 2009), as well as a parameter scaling the entire ODE governing [Ca^2+^]_i_ (distributed evenly between 0 and 1). All other parameter values were preserved from previous modeling studies (Vasalou and Henson, 2010; Vasalou et al., 2011; Kingsbury et al., 2016).

For simulations in which cells were coupled together, the GABA network topology was designed (Kingsbury et al., 2016) to conform to experimental results in which GABA connections were mapped using high-density multi-electrode arrays (Freeman et al., 2013). As described in Kingsbury et al. (2016), the VIP network was taken as a subset of the GABA network by assigning a random variable to each cell that removed outgoing VIP connections from 80% of the network. For dispersed cells, neurons did not receive any VIP or GABA signals, whereas in the coupled network, all neurons were forced to have at least one incoming VIP signal. No VIP or GABA signals were received during simulated TTX application; this was achieved by setting firing rates to zero and increasing the values of parameters governing GABA and VIP degradation (*n_dGABA_* and *n_dVIP_*, respectively) from 0.2 to 1.0.

## Results

### In dispersed SCN neurons, both PER2 and [Ca^2+^]_i_ rhythms are cell autonomous, but the strength of [Ca^2+^]_i_ rhythmicity is limited by PER2 rhythmicity

To study whether [Ca^2+^]_i_ rhythms are cell autonomous or require a rhythmic SCN neuronal network, we measured [Ca^2+^]_i_ in SCN neurons dissociated from PER2::LUC mice and maintained in dispersed cultures. Both PER2 and [Ca^2+^]_i_ dynamics were monitored by imaging in single neurons (Fig. 1*A*). Cells were manually tracked in imaging data over several days (4.7–7.8 d). Rhythmicity of both PER2 expression and [Ca^2+^]_i_ were analyzed for 87 cells in 3 cultures over a 4-d window (Fig. 1). Strength of rhythmicity (RI) was assessed by autocorrelation, and the presence of statistically significant rhythmicity was assessed by the PSD-regression test for both PER2 and [Ca^2+^]_i_, as described in Materials and Methods. There was no statistical correlation between GCaMP3 expression level and [Ca^2+^]_i_ RI. Even cells with the lowest GCaMP3 expression still exhibited robust circadian rhythm, suggesting that weak expression of CCaMP3 was not responsible for lack of rhythmicity in other cells.

Most cells (80%) had robust circadian rhythms of PER2 expression, as reported previously (Liu et al., 2007; Webb et al., 2009; Ko et al., 2010; Fig. 1*C*, *D*). [Ca^2+^]_i_ rhythms, like PER2 rhythms, were independently phased across cells, and therefore cell autonomous. However, only 40% of dispersed cells had significant [Ca^2+^]_i_ rhythms; even in cells with rhythmic PER2, only 46% had significant [Ca^2+^]_i_ rhythms (Fig. 1*B*, *D*). In contrast to the nearly sinusoidal PER2 waveforms, [Ca^2+^]_i_ rhythm waveforms were more variable, often showing spike-like waveforms with narrow peaks (Fig. 1*C*). All cells with significant [Ca^2+^]_i_ rhythms also had significant PER2 rhythms, except for three cells with marginal rhythmicity values (Fig. 1*F*). Peaks of [Ca^2+^]_i_ preceded peaks of PER2 by an average of 7.3 ± 2.2 h (circular mean ± SD, *n* = 32; Fig. 1*E*).

In dispersed SCN neurons, the RI value for [Ca^2+^]_i_ rarely exceeded that for PER2 (Fig. 1*F*). To better understand the relationship of PER2 and [Ca^2+^]_i_ rhythmicity, we ran simulations of dispersed neurons in a mathematical model that included both TTFL and electrophysiology. Three parameters influencing the period and amplitude of TTFL and one parameter influencing [Ca^2+^]_i_ concentration were independently and randomly modulated in 400 dispersed neurons (Vasalou and Henson, 2010), as described in Materials and Methods. Similar to experimental data (Fig. 1*F*), the amplitude of [Ca^2+^]_i_ was limited by the amplitude of PER (Fig. 1*G*). Two mechanisms encoded in the model enabled it to achieve behavior similar to that observed experimentally (Fig. 1*G*): (1) [Ca^2+^]_i_ rhythms were dependent on TTFL rhythms, and (2) TTFL rhythms persisted at baseline levels even in the absence of [Ca^2+^]_i_ oscillations (To et al., 2007).

### A rhythmic SCN neuronal network reinforces both PER2 and [Ca^2+^]_i_ rhythmicity

To examine the effects of the SCN network on PER2 and [Ca^2+^]_i_ rhythmicity, we compared the rhythmicity of single SCN neurons oscillating independently in dispersed cell cultures to those oscillating synchronously in slice cultures. Because we sliced 250-μm coronal sections, the SCN was usually divided into two slices (Noguchi and Watanabe, 2008). The slice containing a larger part of the SCN was used for imaging. Cells located in various parts of SCN slices were evenly selected and manually tracked in imaging data over several days (4.7–6.8 d). Both PER2 and [Ca^2+^]_i_ rhythmicity were analyzed for 45 cells in 3 SCN slices for 4 d (Fig. 2*A–C*). Single-cell PER2 and [Ca^2+^]_i_ rhythms appeared more robust in slice cultures than in dispersed cell cultures (Fig. 2*D*). In SCN slices, as in dispersed cells, [Ca^2+^]_i_ rhythm waveforms were more variable than PER2 rhythm waveforms. In SCN slices, PER2 and [Ca^2+^]_i_ patterns were rhythmic in 100% and 71% of cells, respectively (PSD-regression test, *p* < 0.05). Using RI as a measure of rhythm strength, single-cell PER2 and [Ca^2+^]_i_ rhythms were both significantly stronger in slices than in dispersed cultures (*p* < 0.05, mixed effect model; Fig. 2*E*). To measure coherence between PER2 and [Ca^2+^]_i_ waveforms, cross-correlations were performed for both dispersed cells and cells in slices. The cross-correlation values were significantly higher for cells in slices (Fig. 2*F*, *p* < 0.05, mixed effect model), suggesting higher similarity between PER2 and [Ca^2+^]_i_ waveforms or improved rhythmicity. Peaks of [Ca^2+^]_i_ preceded peaks of PER2 by an average of 4.8 ± 2.6 h (circular mean ± SD, *n* = 32; Fig. 2*G*), which was significantly different from dispersed cells (7.3 ± 2.2 h; circular mean ± SD, *n* = 32; *p* < 0.01, Watson–Williams test; Fig. 1*E*). Compared with dispersed cells, cells in slices also exhibited a significantly larger variability of this difference between PER2 and [Ca^2+^]_i_ peak time (p < 0.05, Wallraff’s nonparametric test of circular homoscedasticity).

### Spatiotemporal relationships between PER2 and [Ca^2+^]_i_ rhythms

To analyze spatiotemporal patterns of PER2 and [Ca^2+^]_i_ activation in the SCN and how the neuronal network may influence those patterns, peak times of PER2 and [Ca^2+^]_i_ across the SCN were computationally detected in five SCN slices. Cell-like regions exhibiting significant rhythmicity in either PER2 or [Ca^2+^]_i_ are depicted as small circles. Spatiotemporal patterns of PER2 and [Ca^2+^]_i_ peaks and phase difference between PER2 and [Ca^2+^]_i_ peaks in all five slices are shown in Fig. 2*H–K*. Generally, peak times of PER2 occurred earlier in the dorsomedial region and later in the ventrolateral region, as reported in previous studies (Evans et al., 2011b; Foley et al., 2011; Fig. 2*H*). [Ca^2+^]_i_ and PER2 phases generally showed a similar spatial pattern (Fig. 2*H–J*). Phase differences between PER2 and [Ca^2+^]_i_ were relatively uniform across SCN, with [Ca^2+^]_i_ peaks preceding PER2 peaks by ∼4–6 h (Fig. 2*K*). Phase relationships between PER2 and [Ca^2+^]_i_ peaks in each slice were stable during the entire 3- to 7-d durations of imaging experiments.

A minority of cells in the dorsomedial region expressed very different phase relationships, including [Ca^2+^]_i_ peaks antiphase to or lagging PER2 peaks. These distinct phase differences were caused by changes in [Ca^2+^]_i_ phasing rather than PER2 phasing (Fig. 2*H–K*). We hypothesized that this unusual phase relationship might arise from a difference in rostrocaudal position of the slice within the SCN. Additional SCN slices containing either a rostral or a caudal tip of the SCN (Noguchi and Watanabe, 2008), were imaged. However, unusual phasing was not observed in these extreme rostral (*n* = 1) or caudal (*n* = 3) slices.

### Synchronous SCN network electrical activity reinforces rhythmic PER2 expression

To determine whether reinforcement of PER2 or [Ca^2+^]_i_ rhythms requires a rhythmic, synchronized SCN network, we compared effects of TTX (blocking neuronal firing) on rhythmicity in slice and dispersed cultures. Both PER2 and [Ca^2+^]_i_ rhythmicity were analyzed for 69 SCN neurons in 5 slices and 45 SCN neurons in 3 dispersed cell cultures. Cultures were imaged in control medium for 3–4 d, then in medium containing 1 µM TTX for 3.0 d, and finally in fresh control medium for 3.8 d.

For SCN neurons in slice cultures, on average, TTX significantly decreased PER2 RI to 70% of the value before TTX, and refreshing medium resulted in partial recovery to 84% of the pre-TTX control value (Fig. 3*A*, *C*). TTX had more variable effects on [Ca^2+^]_i_ rhythms: e.g., no effect or loss of rhythmicity. On average, TTX decreased [Ca^2+^]_i_ RI to 60% of the value before TTX, and refreshing medium resulted in recovery to 95% of the pre-TTX control value (Fig. 3*A*, *C*). TTX also significantly reduced average PER2 expression to 42% of the value before TTX, and refreshing medium restored it to a level similar to that before TTX, consistent with previous reports (Yamaguchi et al., 2003; Fig. 3*A*, *D*). Similarly, the coherence between PER2 and [Ca^2+^]_i_ waveforms significantly decreased during TTX application and recovered after refreshing medium (Fig. 3*E*).

For SCN neurons in dispersed cultures, unlike in slice cultures, TTX did not significantly affect PER2 or [Ca^2+^]_i_ RI (Fig. 3*B*, *F*); nor did TTX significantly affect average PER2 expression or coherence (Fig. 3*G*, *H*). For most individual cell traces, TTX had no discernible effect on PER2 rhythmicity (Fig. 3*B*). Just as in slice cultures, TTX had more variable effects on [Ca^2+^]_i_ rhythms: e.g., no effect (Fig. 3*B*), loss of rhythmicity, or gain of rhythmicity. However, such changes in [Ca^2+^]_i_ rhythmicity were also sometimes observed spontaneously in control conditions without TTX, and on average TTX had no significant effect on [Ca^2+^]_i_ RI (Fig. 3*F*).

Ryanodine receptors have been suggested to regulate circadian [Ca^2+^]_i_ release (Ikeda et al., 2003). We applied 100 µM ryanodine to dispersed SCN cultures (*n* = 20 cells, in 2 cultures), but no significant difference was observed in either PER2 or [Ca^2+^]_i_ RI (Fig. 1–3).

### Mathematical models predict that PER and [Ca^2+^]_i_ rhythm amplitude are reduced by TTX in a multicellular SCN network but not in a single SCN neuron

To explore possible reasons that TTX suppressed PER2 and [Ca^2+^]_i_ rhythmicity in SCN cells in slices but not in dispersed cultures, effects of TTX were simulated in both single-cell and multicellular SCN mathematical models (Vasalou and Henson, 2010, 2011; Vasalou et al., 2011). Neuronal firing rate was set to 0 Hz during TTX application, abolishing VIP and GABA signaling. In the multicellular model, desynchronization of individual cells and the absence of VIP receptor activation during TTX application reduced the amplitude of PER and [Ca^2+^]_i_ oscillations (Fig. 3*I*). In contrast, in the single-cell model, there is no VIP or GABA intercellular signaling even at baseline, in the absence of TTX; consequently, applying TTX had no effect on amplitude of PER and [Ca^2+^]_i_ oscillations (Fig. 3*J*).

### [Ca^2+^]_i_ rhythmicity requires an intact transcriptional clock

To test whether clock gene rhythms are necessary to generate [Ca^2+^]_i_ rhythms in SCN neurons, we imaged [Ca^2+^]_i_ rhythms in SCN slice cultures from mice deficient in the essential clock gene *Bmal1* (Bunger et al., 2000; Ko et al., 2010). Cells were manually tracked in imaging data (4.9–6.7 d). Both PER2 and [Ca^2+^]_i_ rhythms were analyzed for 35 cells in 4 slice cultures for 4 d. Both PER2 and [Ca^2+^]_i_ showed stochastic fluctuations rather than constant levels or circadian rhythms (Fig. 4*A*). PER2 RI, [Ca^2+^]_i_ RI, and coherence between PER2 and [Ca^2+^]_i_ traces were significantly and dramatically decreased compared with WT controls (45 cells in 3 SCN slices; Fig. 4*B*, *C*).

## Discussion

To study the causal relationships among SCN clock gene rhythms, [Ca^2+^]_i_ rhythms, neuronal firing rhythms, and the SCN neuronal network, we simultaneously imaged single-cell PER2 and [Ca^2+^]_i_ rhythmicity in both SCN slice and dispersed cell cultures. Our results demonstrate that [Ca^2+^]_i_ rhythms in SCN neurons (1) are cell autonomous and do not require a synchronized network (Fig. 1), yet (2) are reinforced by the rhythmic neuronal network in SCN slices (Fig. 2), (3) do not require circadian rhythms of neuronal firing (Fig. 3), (4) have flexible phasing modulated by the neuronal network (Fig. 2), and (5) require an intact TTFL (Fig. 4).

In dispersed SCN neurons, circadian rhythms of spontaneous firing or PER2 expression free-run independently with their own intrinsic periods (Welsh et al., 1995; Webb et al., 2009), establishing that firing and PER2 rhythms are cell autonomous. The presence of [Ca^2+^]_i_ rhythms in independently oscillating dispersed SCN neurons, as shown here (Fig. 1), establishes that [Ca^2+^]_i_ rhythms are also cell-autonomous.

Many dispersed SCN neurons did not show [Ca^2+^]_i_ rhythms yet exhibited robust PER2 rhythms. The strength of [Ca^2+^]_i_ rhythmicity was always limited by PER2 rhythmicity, i.e., never clearly stronger than PER2 rhythmicity in a given cell. These experimental results are consistent with mathematical simulations, in which [Ca^2+^]_i_ rhythms were not necessary to generate clock gene rhythms in single cells. We propose that this analysis method, plotting a rhythmicity strength of two parameters, can be used to test the causality of two rhythmic parameters. Cytoplasmic Ca^2+^ is, of course, necessary for cell survival and various physiologic functions including circadian rhythm generation (Lundkvist et al., 2005; Colwell, 2011). However, it appears that only tonic levels of [Ca^2+^]_i_ (and not circadian rhythmicity of [Ca^2+^]_i_) are necessary for these processes. Our data suggest that [Ca^2+^]_i_ oscillation works as a clock-controlled messenger to regulate circadian cellular functions rather than as a clock component to generate circadian rhythms.

Both PER2 and [Ca^2+^]_i_ rhythms, but especially the latter, were greatly strengthened in SCN slice cultures relative to dispersed cultures, suggesting that the SCN neuronal network amplifies [Ca^2+^]_i_ rhythms. Coherence between PER2 and [Ca^2+^]_i_ rhythms was also improved in slice cultures, suggesting that control of PER2 over [Ca^2+^]_i_ is stronger in slices than in dispersed cells. We cannot, however, rule out the possibility that improved rhythmicity in SCN slices was at least partly artifactual, owing to scattered light from neighboring cells smoothing the rhythm wave form and reducing noise. This concern could be addressed by future experiments restricting [Ca^2+^]_i_ reporter expression to only a few cells per SCN slice, e.g., by gene gun (Ikeda et al., 2003).

Several lines of studies consistently suggested that TTX application reduces *Per1* and *Per2* mRNA and protein levels in cultured SCN slices (Yamaguchi et al., 2003; Webb et al., 2009). However, previous studies have found variable effects of TTX on [Ca^2+^]_i_ rhythms in SCN slices (Colwell, 2000a; Ikeda et al., 2003; Enoki et al., 2012; Hong et al., 2012; Brancaccio et al., 2013). In our SCN slice cultures, TTX decreased both PER2 and [Ca^2+^]_i_ rhythmicity, as well as PER2 expression levels. However, interestingly, in dispersed cell cultures, which already lack coupling sufficient to synchronize cellular rhythms, TTX did not significantly affect either PER2 or [Ca^2+^]_i_ rhythms. Mathematical models of single-cell and multicellular SCN correctly simulated these distinct TTX effects, indicating that acute reduction of *Pers* by TTX within a day are caused by loss of network reinforcement. They also explain why TTX induces nonphotic-type phase shifts in neonatal rat SCN slices (Noguchi and Watanabe, 2005) but not in dispersed cells (Welsh et al., 1995).

On average, [Ca^2+^]_i_ peaked ∼7.3 h before PER2 for single SCN neurons in dispersed cell cultures, and ∼4.8 h before PER2 for single SCN neurons in slice cultures. These results are similar to the 7.2-h phase difference observed previously in SCN slices (Brancaccio et al., 2013). In our study, the average phase difference between PER2 and [Ca^2+^]_i_ rhythms in single cells was significantly different for SCN slices versus dispersed cell cultures (Figs. 1*E* and 2*G*), with a wider distribution of phase differences among cells in slices than among dispersed cells. These results suggest that the SCN neuronal network modulates the phase relationship between PER2 and [Ca^2+^]_i_ rhythms.

More detailed computational spatiotemporal peak time analysis showed that [Ca^2+^]_i_ peaked ∼4–6 h before PER2 in most cells in SCN slices (Fig. 2*H–S*), as suggested in previous work (Enoki et al., 2012; Brancaccio et al., 2013). However, in some slices, a small dorsal SCN region stably maintained [Ca^2+^]_i_ peak time nearly antiphasic to the PER2 peak (Fig. 2*J*). These divergent groups of cells were not seen in rostral or caudal slices. Possibly, the divergent phase relationship may occur selectively in slices containing VIP-positive cells, which are more abundant in the central part of the SCN (Abrahamson and Moore, 2001; Noguchi and Watanabe, 2008).

Compared with the stable sinusoidal waveform of PER2 rhythms, [Ca^2+^]_i_ rhythm waveforms were more variable. For example, [Ca^2+^]_i_ rhythms showed narrow spike type waveforms (Figs. 1*C* and 2*D* ), sinusoidal waveforms, two daily [Ca^2+^]_i_ peaks, or arrhythmicity (Fig. 1*B*). The diverse phases and waveforms of [Ca^2+^]_i_ rhythms in dispersed SCN neurons suggest that [Ca^2+^]_i_ rhythms can be modulated not only by the intercellular neuronal network, but also by intracellular modulators linking the TTFL to [Ca^2+^]_i_. Such intracellular mechanisms could involve store-operated Ca^2+^ entry (SOCE; Ikeda et al., 2003; Shen et al., 2011). Further investigations are necessary to determine the specific mechanisms and possible significance of variable [Ca^2+^]_i_ waveforms and variable phase relationships between PER2 and [Ca^2+^]_i_ rhythms.

It has been reported that circadian rhythms of [Ca^2+^]_i_ and electrical activity can persist despite treatment with *Per1* + *Per2* antisense oligonucleotides, which significantly reduced PER immunoreactivity (Sugiyama et al., 2004). Thus, we considered the possibility that [Ca^2+^]_i_ rhythms could be driven by TTFL-independent mechanisms. In this study, we showed that [Ca^2+^]_i_ rhythms were definitively abolished in *Bmal1^–/–^* SCN slices, in which PER2 is also nonrhythmic. This is consistent with the hypothesis that an intact TTFL drives [Ca^2+^]_i_ rhythms in SCN neurons (Enoki et al., 2017a). In the previous experiment using *Per1* + *Per2* antisense oligonucleotides (Sugiyama et al., 2004), [Ca^2+^]_i_ rhythms may have been rescued by a few cells in which the TTFL was not completely suppressed by antisense oligonucleotides (Liu et al., 2007). In some cases, *Bmal1^–/–^* SCN slices (but not dispersed cells) have been observed to generate stochastic PER2 oscillations with variable periods overlapping the circadian range (Ko et al., 2010). We did not observe such unstable oscillations in this study, possibly because of toxicity of viral vectors or [Ca^2+^]_i_ buffering by GCaMP3 (Looger and Griesbeck, 2012).

Studies in SCN slices have found that Ca^2+^ rhythms depend on Gq signaling (Brancaccio et al., 2013) and ryanodine-sensitive intracellular Ca^2+^ stores (Ikeda et al., 2003), but not on gap junctions (Enoki et al., 2012). Ryanodine receptors have been shown to modulate temporal [Ca^2+^]_i_ release in the mouse SCN and to be necessary for rat behavioral rhythm output (Mercado et al., 2009; Aguilar-Roblero et al., 2016). In this study, ryanodine had little effect on circadian rhythms of [Ca^2+^]_i_ or PER2. Differences in experimental conditions, such as developmental stage and reporters, might have led to different [Ca^2+^]_i_ responses, but, our report is consistent with previous work in finding that ryanodine does not affect PER2 rhythms (Aguilar-Roblero et al., 2016). Possible mechanisms by which clock genes control spontaneous firing of SCN neurons include regulation of ion channels at the transcriptional level (e.g., BK calcium-activated potassium channels; Meredith et al., 2006), via cAMP [e.g., hyperpolarization-activated, cyclic nucleotide-gated (HCN) ion channels; O’Neill et al., 2008], or via redox status (Wang et al., 2012). However, the mechanism by which the TTFL exerts rhythmic control over [Ca^2+^]_i_ release from intracellular stores is completely unknown. SCN microarray studies reveal circadian oscillation of several interesting Ca^2+^ related genes (e.g., calreticulin and calumenin, which are Ca^2+^-binding proteins located in ER, and NCX1, which is a sodium/calcium exchanger expressed in plasma membrane and mitochondria; Panda et al., 2002; Jung and Kim, 2004; Barbuti and DiFrancesco, 2008). Further investigation may reveal interesting connections between the TTFL and Ca^2+^ regulatory proteins.

In summary, in this study, we demonstrate autonomy of circadian [Ca^2+^]_i_ rhythms in dispersed SCN neurons, SCN network reinforcement of both PER2 and [Ca^2+^]_i_ rhythms and modulation of the phase relationship between the two rhythms, and dependence of SCN [Ca^2+^]_i_ rhythms on the transcriptional circadian clock but not on neuronal firing within individual cells. How the transcriptional circadian clock drives [Ca^2+^]_i_ rhythms, and how SCN neuronal networks modulate [Ca^2+^]_i_ rhythm phase relative to PER2, are intriguing questions for future study. Much work remains to be done to elucidate the complete mechanism of the SCN circadian clock, including cytosolic signaling pathways and intercellular networks.

## Synthesis

Reviewing Editor: Cheryl Sisk, Michigan State University Decisions are customarily a result of the Reviewing Editor and the peer reviewers coming together and discussing their recommendations until a consensus is reached. When revisions are invited, a fact-based synthesis statement explaining their decision and outlining what is needed to prepare a revision will be listed below. Note: If this manuscript was transferred from JNeurosci and a decision was made to accept the manuscript without peer review, a brief statement to this effect will instead be what is listed below.

Authors were responsive to the JN reviewers.

## References

[B1] Abrahamson EE, Moore RY (2001) Suprachiasmatic nucleus in the mouse: retinal innervation, intrinsic organization and efferent projections. Brain Res 916:172–191. 11597605 10.1016/s0006-8993(01)02890-6

[B2] Aguilar-Roblero R, Quinto D, Baez-Ruiz A, Chavez JL, Belin AC, Diaz-Munoz M, Michel S, Lundkvist G (2016) Ryanodine-sensitive intracellular Ca2+ channels are involved in the output from the SCN circadian clock. Eur J Neurosci 44:2504–2514. 27529310 10.1111/ejn.13368 PMC5053303

[B3] Barbuti A, DiFrancesco D (2008) Control of cardiac rate by “funny” channels in health and disease. Ann N Y Acad Sci 1123:213–223. 10.1196/annals.1420.024 18375593

[B4] Bates D, Mächler M, Bolker BM, Walker SC (2015) Fitting linear mixed-effects models using lme4. J Stat Softw 67:1–48. 10.18637/jss.v067.i01

[B5] Berens P (2009) CircStat: a MATLAB toolbox for circular statistics. J Stat Softw 31:1–21. 10.18637/jss.v031.i10

[B6] Brancaccio M, Maywood ES, Chesham JE, Loudon AS, Hastings MH (2013) A Gq-Ca2+ axis controls circuit-level encoding of circadian time in the suprachiasmatic nucleus. Neuron 78:714–728. 10.1016/j.neuron.2013.03.011 23623697 PMC3666084

[B7] Brockhoff PB, Amorim ID, Kuznetsova A, Bech S, de Lima RR (2016) Delta-tilde interpretation of standard linear mixed model results. Food Qual Prefer 49:129–139. 10.1016/j.foodqual.2015.11.009

[B8] Bunger MK, Wilsbacher LD, Moran SM, Clendenin C, Radcliffe LA, Hogenesch JB, Simon MC, Takahashi JS, Bradfield CA (2000) Mop3 is an essential component of the master circadian pacemaker in mammals. Cell 103:1009–1017. 11163178 10.1016/s0092-8674(00)00205-1 PMC3779439

[B9] Colwell CS (2000a) Circadian modulation of calcium levels in cells in the suprachiasmatic nucleus. Eur J Neurosci 12:571–576. 10712636 10.1046/j.1460-9568.2000.00939.x PMC4353598

[B10] Colwell CS (2000b) Rhythmic coupling among cells in the suprachiasmatic nucleus. J Neurobiol 43:379–388. 10861563 10.1002/1097-4695(20000615)43:4<379::aid-neu6>3.0.co;2-0 PMC2577317

[B11] Colwell CS (2011) Linking neural activity and molecular oscillations in the SCN. Nat Rev Neurosci 12:553–569. 10.1038/nrn3086 21886186 PMC4356239

[B12] Enoki R, Ono D, Kuroda S, Honma S, Honma KI (2017a) Dual origins of the intracellular circadian calcium rhythm in the suprachiasmatic nucleus. Sci Rep 7:41733 28155916 10.1038/srep41733 PMC5290527

[B13] Enoki R, Oda Y, Mieda M, Ono D, Honma S, Honma KI (2017b) Synchronous circadian voltage rhythms with asynchronous calcium rhythms in the suprachiasmatic nucleus. Proc Natl Acad Sci U S A 114:E2476–E2485. 28270612 10.1073/pnas.1616815114 PMC5373333

[B14] Enoki R, Kuroda S, Ono D, Hasan MT, Ueda T, Honma S, Honma K (2012) Topological specificity and hierarchical network of the circadian calcium rhythm in the suprachiasmatic nucleus. Proc Natl Acad Sci U S A 109:21498–21503. 10.1073/pnas.1214415110 23213253 PMC3535646

[B15] Evans JA, Elliott JA, Gorman MR (2011a) Dim nighttime illumination interacts with parametric effects of bright light to increase the stability of circadian rhythm bifurcation in hamsters. Chronobiol Int 28:488–496. 21797777 10.3109/07420528.2011.591952

[B16] Evans JA, Leise TL, Castanon-Cervantes O, Davidson AJ (2011b) Intrinsic regulation of spatiotemporal organization within the suprachiasmatic nucleus. PloS One 6:e15869 21249213 10.1371/journal.pone.0015869 PMC3017566

[B17] Foley NC, Tong TY, Foley D, Lesauter J, Welsh DK, Silver R (2011) Characterization of orderly spatiotemporal patterns of clock gene activation in mammalian suprachiasmatic nucleus. Eur J Neurosci 33:1851–1865. 10.1111/j.1460-9568.2011.07682.x 21488990 PMC3423955

[B18] Freeman GM, Jr., Krock RM, Aton SJ, Thaben P, Herzog ED (2013) GABA networks destabilize genetic oscillations in the circadian pacemaker. Neuron 78:799–806. 10.1016/j.neuron.2013.04.003 23764285 PMC3683151

[B19] Harrisingh MC, Wu Y, Lnenicka GA, Nitabach MN (2007) Intracellular Ca2+ regulates free-running circadian clock oscillation in vivo. J Neurosci 27:12489–12499. 10.1523/JNEUROSCI.3680-07.2007 18003827 PMC6673328

[B20] Hong JH, Jeong B, Min CH, Lee KJ (2012) Circadian waves of cytosolic calcium concentration and long-range network connections in rat suprachiasmatic nucleus. Eur J Neurosci 35:1417–1425. 10.1111/j.1460-9568.2012.08069.x 22501027

[B21] Ikeda M, Sugiyama T, Wallace CS, Gompf HS, Yoshioka T, Miyawaki A, Allen CN (2003) Circadian dynamics of cytosolic and nuclear Ca2+ in single suprachiasmatic nucleus neurons. Neuron 38:253–263. 12718859 10.1016/s0896-6273(03)00164-8

[B22] Johnson CH, Knight MR, Kondo T, Masson P, Sedbrook J, Haley A, Trewavas A (1995) Circadian oscillations of cytosolic and chloroplastic free calcium in plants. Science 269:1863–1865. 7569925 10.1126/science.7569925

[B23] Jung DH, Kim DH (2004) Characterization of isoforms and genomic organization of mouse calumenin. Gene 327:185–194. 10.1016/j.gene.2003.10.014 14980715

[B24] Kingsbury NJ, Taylor SR, Henson MA (2016) Inhibitory and excitatory networks balance cell coupling in the suprachiasmatic nucleus: a modeling approach. J Theor Biol 397:135–144. 10.1016/j.jtbi.2016.02.039 26972478 PMC4828267

[B25] Ko CH, Yamada YR, Welsh DK, Buhr ED, Liu AC, Zhang EE, Ralph MR, Kay SA, Forger DB, Takahashi JS (2010) Emergence of noise-induced oscillations in the central circadian pacemaker. PLoS Biology 8:e1000513. 10.1371/journal.pbio.1000513 20967239 PMC2953532

[B26] Leise TL, Wang CW, Gitis PJ, Welsh DK (2012) Persistent cell-autonomous circadian oscillations in fibroblasts revealed by six-week single-cell imaging of PER2::LUC bioluminescence. PloS One 7:e33334. 10.1371/journal.pone.0033334 22479387 PMC3315561

[B27] Leloup J-C, Goldbeter A (2003) Toward a detailed computational model for the mammalian circadian clock. Proc Natl Acad Sci U S A 100:7051–7056. 10.1073/pnas.1132112100 12775757 PMC165828

[B28] Lewandoski M, Wassarman KM, Martin GR (1997) Zp3-cre, a transgenic mouse line for the activation or inactivation of loxP-flanked target genes specifically in the female germ line. Curr Biol 7:148–151. 9016703 10.1016/s0960-9822(06)00059-5

[B29] Liang X, Holy TE, Taghert PH (2016) Synchronous Drosophila circadian pacemakers display nonsynchronous Ca(2)(+) rhythms in vivo. Science 351:976–981. 10.1126/science.aad3997 26917772 PMC4836443

[B30] Liu AC, Welsh DK, Ko CH, Tran HG, Zhang EE, Priest AA, Buhr ED, Singer O, Meeker K, Verma IM, Doyle FJ, 3rd, Takahashi JS, Kay SA (2007) Intercellular coupling confers robustness against mutations in the SCN circadian clock network. Cell 129:605–616. 10.1016/j.cell.2007.02.047 17482552 PMC3749832

[B31] Looger LL, Griesbeck O (2012) Genetically encoded neural activity indicators. Curr Opin Neurobiol 22:18–23. 10.1016/j.conb.2011.10.024 22104761

[B32] Lundkvist GB, Kwak Y, Davis EK, Tei H, Block GD (2005) A calcium flux is required for circadian rhythm generation in mammalian pacemaker neurons. J Neurosci 25:7682–7686. 10.1523/JNEUROSCI.2211-05.2005 16107654 PMC6725395

[B33] Maywood ES, Chesham JE, O’Brien JA, Hastings MH (2011) A diversity of paracrine signals sustains molecular circadian cycling in suprachiasmatic nucleus circuits. Proc Natl Acad Sci U S A 108:14306–14311. 10.1073/pnas.1101767108 21788520 PMC3161534

[B34] Mercado C, Díaz-Muñoz M, Alamilla J, Valderrama K, Morales-Tlalpan V, Aguilar-Roblero R (2009) Ryanodine-sensitive intracellular Ca2+ channels in rat suprachiasmatic nuclei are required for circadian clock control of behavior. J Biol Rhythms 24:203–210. 10.1177/0748730409333354 19465697

[B35] Meredith AL, Wiler SW, Miller BH, Takahashi JS, Fodor AA, Ruby NF, Aldrich RW (2006) BK calcium-activated potassium channels regulate circadian behavioral rhythms and pacemaker output. Nat Neurosci 9:1041–1049. 10.1038/nn1740 16845385 PMC2909323

[B36] Mohawk JA, Takahashi JS (2011) Cell autonomy and synchrony of suprachiasmatic nucleus circadian oscillators. Trends Neurosci 34:349–358. 10.1016/j.tins.2011.05.003 21665298 PMC3775330

[B37] Noguchi T, Watanabe K (2005) Tetrodotoxin resets the clock. Eur J Neurosci 21:3361–3367. 10.1111/j.1460-9568.2005.04180.x 16026473

[B38] Noguchi T, Watanabe K (2008) Regional differences in circadian period within the suprachiasmatic nucleus. Brain Res 1239:119–126. 10.1016/j.brainres.2008.08.082 18801342

[B39] O’Neill JS, Maywood ES, Chesham JE, Takahashi JS, Hastings MH (2008) cAMP-dependent signaling as a core component of the mammalian circadian pacemaker. Science 320:949–953. 18487196 10.1126/science.1152506 PMC2735813

[B40] Obrietan K, Impey S, Smith D, Athos J, Storm DR (1999) Circadian regulation of cAMP response element-mediated gene expression in the suprachiasmatic nuclei. J Biol Chem 274:17748–17756. 10364217 10.1074/jbc.274.25.17748

[B41] Panda S, Antoch MP, Miller BH, Su AI, Schook AB, Straume M, Schultz PG, Kay SA, Takahashi JS, Hogenesch JB (2002) Coordinated transcription of key pathways in the mouse by the circadian clock. Cell 109:307–320. 12015981 10.1016/s0092-8674(02)00722-5

[B42] Shen WW, Frieden M, Demaurex N (2011) Remodelling of the endoplasmic reticulum during store-operated calcium entry. Biol Cell 103:365–380. 10.1042/BC20100152 21736554

[B43] Silver R, LeSauter J, Tresco PA, Lehman MN (1996) A diffusible coupling signal from the transplanted suprachiasmatic nucleus controlling circadian locomotor rhythms. Nature 382:810–813. 10.1038/382810a0 8752274

[B44] Storch KF, Paz C, Signorovitch J, Raviola E, Pawlyk B, Li T, Weitz CJ (2007) Intrinsic circadian clock of the mammalian retina: importance for retinal processing of visual information. Cell 130:730–741. 10.1016/j.cell.2007.06.045 17719549 PMC2040024

[B45] Sugiyama T, Yoshioka T, Ikeda M (2004) mPer2 antisense oligonucleotides inhibit mPER2 expression but not circadian rhythms of physiological activity in cultured suprachiasmatic nucleus neurons. Biochem Biophys Res Commun 323:479–483. 10.1016/j.bbrc.2004.08.126 15369776

[B46] Tian L, Hires SA, Mao T, Huber D, Chiappe ME, Chalasani SH, Petreanu L, Akerboom J, McKinney SA, Schreiter ER, Bargmann CI, Jayaraman V, Svoboda K, Looger LL (2009) Imaging neural activity in worms, flies and mice with improved GCaMP calcium indicators. Nat Methods 6:875–881. 10.1038/nmeth.1398 19898485 PMC2858873

[B47] Tischkau SA, Mitchell JW, Tyan S-H, Buchanan GF, Gillette MU (2003) Ca2+/cAMP response element-binding protein (CREB)-dependent activation of Per1 is required for light-induced signaling in the suprachiasmatic nucleus circadian clock. J Biol Chem 278:718–723. 10.1074/jbc.M209241200 12409294

[B48] To T-L, Henson MA, Herzog ED, Doyle IIIFJ (2007) A molecular model for intercellular synchronization in the mammalian circadian clock. Biophys J 92:3792–3803. 10.1529/biophysj.106.094086 17369417 PMC1868999

[B49] Vasalou C, Henson MA (2010) A multiscale model to investigate circadian rhythmicity of pacemaker neurons in the suprachiasmatic nucleus. PLoS Comput Biol 6:e1000706. 10.1371/journal.pcbi.1000706 20300645 PMC2837390

[B50] Vasalou C, Henson MA (2011) A multicellular model for differential regulation of circadian signals in the core and shell regions of the suprachiasmatic nucleus. J Theor Biol 288:44–56. 10.1016/j.jtbi.2011.08.010 21871462 PMC3184462

[B51] Vasalou C, Herzog ED, Henson MA (2009) Small-world network models of intercellular coupling predict enhanced synchronization in the suprachiasmatic nucleus. J Biol Rhythms 24:243–254. 10.1177/0748730409333220 19465701 PMC2819153

[B52] Vasalou C, Herzog ED, Henson MA (2011) Multicellular model for intercellular synchronization in circadian neural networks. Biophys J 101:12–20. 10.1016/j.bpj.2011.04.051 21723810 PMC3127187

[B53] Wang TA, Yu YV, Govindaiah G, Ye X, Artinian L, Coleman TP, Sweedler JV, Cox CL, Gillette MU (2012) Circadian rhythm of redox state regulates excitability in suprachiasmatic nucleus neurons. Science 337:839–842. 10.1126/science.1222826 22859819 PMC3490628

[B54] Webb AB, Angelo N, Huettner JE, Herzog ED (2009) Intrinsic, nondeterministic circadian rhythm generation in identified mammalian neurons. Proc Natl Acad Sci U S A 106:16493–16498. 10.1073/pnas.0902768106 19805326 PMC2752526

[B55] Welsh DK, Noguchi T (2012) Cellular bioluminescence imaging. Cold Spring Harb Protoc 2012:pii: pdb.top070607. 10.1101/pdb.top070607 22854570

[B56] Welsh DK, Imaizumi T, Kay SA (2005) Real-time reporting of circadian-regulated gene expression by luciferase imaging in plants and mammalian cells. Methods Enzymol 393:269–288. 10.1016/S0076-6879(05)93011-5 15817294

[B57] Welsh DK, Takahashi JS, Kay SA (2010) Suprachiasmatic nucleus: cell autonomy and network properties. Ann Rev Physiol 72:551–577. 10.1146/annurev-physiol-021909-135919 20148688 PMC3758475

[B58] Welsh DK, Logothetis DE, Meister M, Reppert SM (1995) Individual neurons dissociated from rat suprachiasmatic nucleus express independently phased circadian firing rhythms. Neuron 14:697–706. 7718233 10.1016/0896-6273(95)90214-7

[B59] Welsh DK, Yoo SH, Liu AC, Takahashi JS, Kay SA (2004) Bioluminescence imaging of individual fibroblasts reveals persistent, independently phased circadian rhythms of clock gene expression. Curr Biol 14:2289–2295. 10.1016/j.cub.2004.11.057 15620658 PMC3777438

[B60] Yamaguchi S, Isejima H, Matsuo T, Okura R, Yagita K, Kobayashi M, Okamura H (2003) Synchronization of cellular clocks in the suprachiasmatic nucleus. Science 302:1408–1412. 10.1126/science.1089287 14631044

[B61] Yoo SH, Yamazaki S, Lowrey PL, Shimomura K, Ko CH, Buhr ED, Siepka SM, Hong HK, Oh WJ, Yoo OJ, Menaker M, Takahashi JS (2004) PERIOD2::LUCIFERASE real-time reporting of circadian dynamics reveals persistent circadian oscillations in mouse peripheral tissues. Proc Natl Acad Sci U S A 101:5339–5346. 10.1073/pnas.0308709101 14963227 PMC397382

